# An observational analysis of the impact of indoor residual spraying in two distinct contexts of Burkina Faso

**DOI:** 10.1186/s12936-024-05054-2

**Published:** 2024-08-02

**Authors:** Emily R. Hilton, Gauthier Tougri, Tiécoura Camara, Ardjouma Pagabelem, Jean Baptiste Ouedraogo, Justin Millar, Djenam Jacob, Adama Kone, Mame Diouf, Allison Belemvire, Sarah Burnett

**Affiliations:** 1 PMI VectorLink Project, PATH, 2201 Westlake Avenue, Suite 200, Seattle, WA 98121 USA; 2 PMI VectorLink Project, Abt Associates, Rockville, MD USA; 3 Programme National de Lutte Contre le Paludisme, Ouagadougou, Burkina Faso; 4 U.S. President’s Malaria Initiative, U.S. Agency for International Development, Ouagadougou, Burkina Faso; 5 U.S. President’s Malaria Initiative, U.S. Agency for International Development, Washington, DC USA

**Keywords:** Indoor residual spraying, Observational analysis, Combined malaria control strategies

## Abstract

**Background:**

Indoor residual spraying (IRS) is a cornerstone malaria control intervention in Burkina Faso. From 2018 to 2021, non-pyrethroid IRS was implemented annually in two regions of Burkina Faso with distinct malaria transmission patterns, concurrently with annual seasonal malaria chemoprevention (SMC), and a mass insecticide-treated net (ITN) distribution in 2019.

**Methods:**

A retrospective quasi-experimental approach was used to evaluate the impact of the 2018, 2020, and 2021 IRS campaigns on routinely reported confirmed malaria case incidence at health facilities. The 2019 campaign was excluded due to lack of data reporting during a health sector strike. Controlled interrupted time series models were fit to detect changes in level and trend in malaria case incidence rates following each IRS campaign when compared to the baseline period 24-months before IRS. IRS districts Solenzo (Sudano-Sahelien climate), and Kampti (tropical climate) were compared with neighbouring control districts and the analyses were stratified by region. Modelled health facility catchment population estimates based on travel time to health facilities and weighted by non-malaria outpatient visits were used as an offset. The study period encompassed July 2016 through June 2022, excluding July 2018 to June 2019.

**Results:**

District-level population and structure coverage achieved by IRS campaigns was greater than 85% in 2018, 2020, and 2021 in Solenzo and Kampti. In Solenzo a significant difference in malaria case incidence rates was detected after the 2018 campaign (IRR = 0.683; 95% CI 0.564–0.827) when compared to the control district. The effect was not detected following the 2020 or 2021 IRS campaigns. In Kampti, estimated malaria incidence rates were between 36 and 38% lower than in the control district following all three IRS campaigns compared to the baseline period.

**Conclusions:**

Implementation of IRS in Kampti, a tropical region of Burkina Faso, appeared to have a consistent significant beneficial impact on malaria case rates. An initial positive impact in Solenzo after the first IRS campaign was not sustained in the successive evaluated IRS campaigns. This study points to a differential effect of IRS in different malaria transmission settings and in combination with ITN and SMC implementation.

**Supplementary Information:**

The online version contains supplementary material available at 10.1186/s12936-024-05054-2.

## Background

Global malaria mortality has declined steadily since 2000. Between 2000 and 2015, the incidence rate of clinical disease nearly halved, although in more recent years it has begun to level off [1]. Vector control interventions are among the most critical tools in the arsenal of strategies against malaria. Recent modelling has estimated that nearly 81% of all malaria cases averted from 2000 to 2015 are attributable to large scale vector control, including indoor residual spraying (IRS) [2]. IRS is a method of coating the walls and other interior surfaces of households with a residual insecticide that will kill mosquitoes that come into contact with these surfaces [3]. However, despite its recognition as a highly effective form of vector control [4] and an integral part of global malaria control and elimination [5], the continued success of IRS is vulnerable to complex external factors. Of great concern is increasing insecticide resistance, especially to pyrethroids [6], the most commonly used class of insecticide for malaria vector control, as well as concerns around increased costs and gaps in the evidence base needed to evaluate the effectiveness of newer non-pyrethroid products. Among the currently available long-lasting pyrethroid alternatives are a microencapsulated formulation of the organophosphate pirimiphos-methyl (PM CS); and clothianidin, a neonicotinoid. There exists strong experimental evidence for the effectiveness of IRS products containing these insecticides [7–10] and both have been recently deployed in IRS campaigns in the Solenzo and Kampti districts of Burkina Faso. However, few studies to date have assessed the impact of clothianidin-based IRS on real-world epidemiological indicators [11], nor the impact of IRS in Sudano-Sahelian settings [12].

In 2020, Burkina Faso accounted for 3% of malaria deaths and 3.4% of malaria cases globally [1], reporting nearly 11.3 million cases nationwide [13]. In 2018, the World Health Organization (WHO) launched the High Burden to High Impact (HBHI) initiative which supports the 11 highest burden countries, including Burkina Faso, in accelerating progress against malaria. A key pillar in the HBHI approach is movement away from a “one size fits all” approach and toward strategies that leverage strategic use of data, including routine health data, to optimize the delivery of malaria control interventions [14]. The U.S. President’s Malaria Initiative (PMI) has also emphasized sub-national tailoring of interventions using evidence from operational research and impact evaluations [15]. There is increasing interest in the use of routine health data to support this type of evidence-generation, given the impracticality of conducting regular large-scale, cross-sectional, community-based surveys. However, the quality of routine health data is dependent on factors including care-seeking behaviour, geographic access to care, availability of diagnostic kits and treatments, and disruptions to data reporting. In 2019, Burkina Faso experienced a strike of health care workers which deliberately interrupted the reporting of routine data for 6 months, resulting in significant gaps [16].

Sub-national tailoring of malaria control packages also requires evidence around which interventions are best layered together to produce maximum impact. Studies that assess the impact of IRS in the presence of other malaria control interventions will help decision-makers understand the impact of mixing interventions in diverse contexts. There is already some evidence that the addition of non-pyrethroid IRS in communities using insecticide-treated nets (ITNs) is associated with reduced malaria prevalence [17, 18], and that combining IRS and seasonal malaria chemoprevention (SMC) has a greater effect on reducing incidence than either intervention alone [19, 20].

Over the past 10 years, Burkina Faso has prioritized reducing the country’s malaria burden and has implemented several large-scale strategies to reduce the incidence of malaria, including mass distribution of ITNs every 3 years since 2010; annual SMC campaigns starting in 2014; and implementation of free healthcare for children under 5 years of age starting in 2016 [21]. Burkina Faso’s 2016 to 2020 malaria national strategic plan included reintroduction of IRS as a key community-level intervention [22]. IRS had previously been implemented as a pilot in Burkina Faso in a single district from 2010 to 2012, but the activity was discontinued due to funding constraints. In 2017 Burkina Faso was selected as a PMI focus country, which enabled the National Malaria Control Programme (NMCP) to implement large-scale IRS campaigns across three health districts starting in 2018. From 2018 to 2021, the PMI VectorLink project conducted yearly IRS campaigns [23], and a previous evaluation of the 2018 IRS campaign in three districts (Solenzo, Kampti, and Kongoussi) found an overall significant impact of IRS on malaria incidence, but did not differentially examine the impact in each district [24].

This study presents a retrospective quasi-experimental time-series (2016–2022) analysis of the epidemiological impact of IRS campaigns in Solenzo and Kampti, Burkina Faso in 2018, 2020, and 2021. The 2019 IRS campaign is not included in the analysis due to a six-month disruption of health facility data reporting in 2019. The analysis relies on routine health data with the aim of expanding the evidence base around the public health impact of IRS campaigns and fostering the use of routine health data for evidence-based decision-making.

## Methods

### Study setting

Burkina Faso is a land-locked country in West Africa sitting between 200 and 750 m above sea level, with slight differences between its lowest and highest elevations. The country is spread across three distinct climate zones: an arid Sahelien climate in the north; a wet tropical Sudanian climate in the southwest; and a warm semi-arid Sudano-Sahelien climate through the middle swathe of the country. Annual rainfall in the northern Sahelien zone is only around 400 mm per year and increases as one moves southwest toward more tropical areas where rainfall typically reaches 1000–1200 mm per year. The southwest is likewise more humid and more densely vegetated than both the Sudano-Sahelien and Sahelien zones [25].

Malaria is endemic throughout Burkina Faso, with seasonal peaks occurring from July through October in most areas. Seasonal transmission patterns are most distinct in the Sahelien and Sudano-Sahelien climate zones and are less pronounced in the tropical southwest (see supplemental material for more details) [22].

**Fig. 1:**
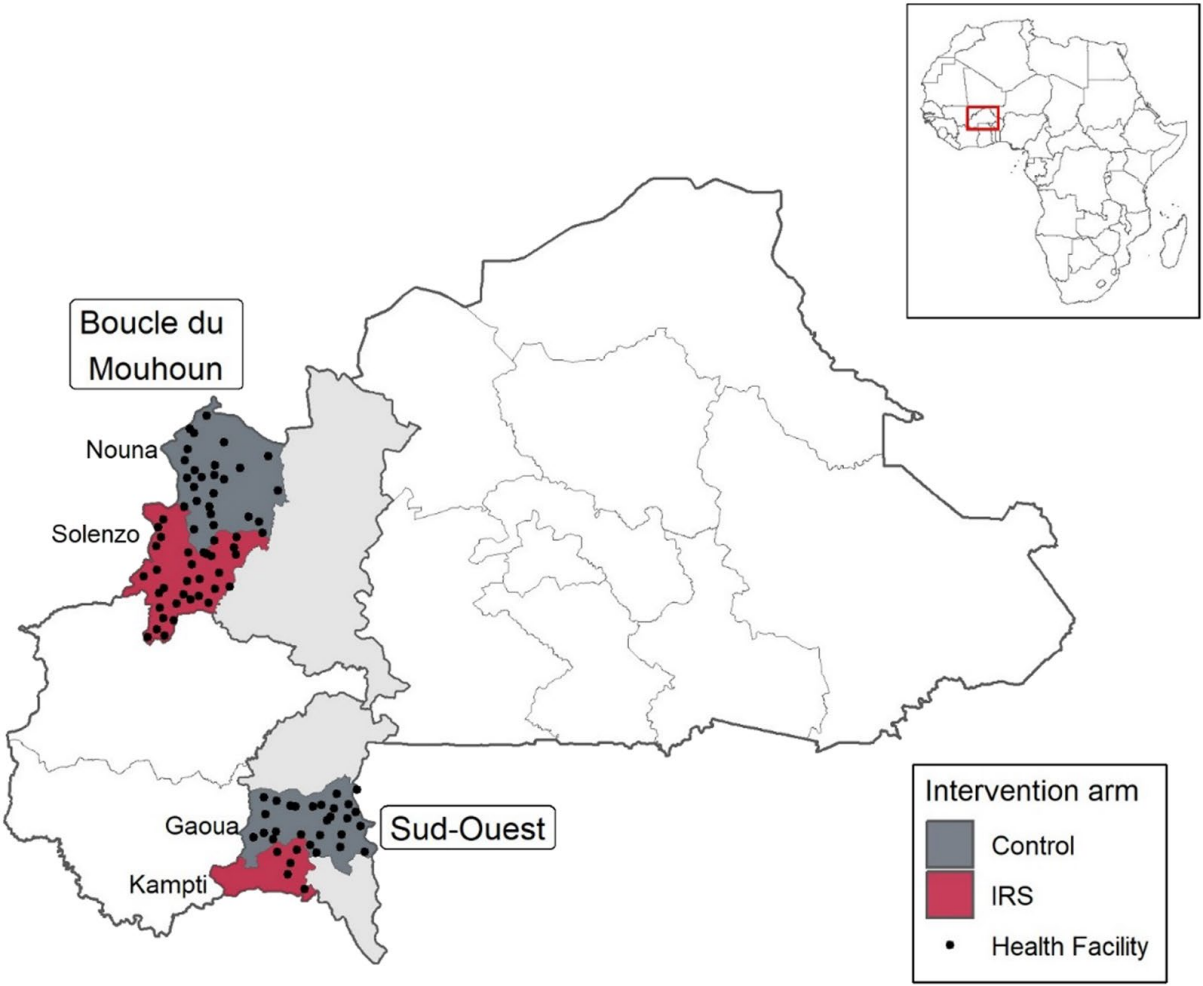
Location of IRS and control districts. Solenzo and Nouna are located in the Boucle du Mouhoun region; Kampti and Gaoua are located in the Sud-Ouest region. Black points represent health facilities that were included in the primary analysis

This study focused on four districts located in two different regions of Burkina Faso: (1) Solenzo and Nouna districts, which are situated in the western Boucle du Mouhoun region in a Sudano-Sahelien climate zone; and (2) Kampti and Gaoua districts, located in the tropical Sud-Ouest region (Fig. 1). Malaria prevalence in children 6–59 months was 23% in 2017 in the Boucle du Mouhoun region. In the Sud-Ouest, malaria prevalence in children was 39% in 2017, the highest in the country [26]. Malaria is the top cause of hospitalization and mortality in Burkina Faso [26].

The parasite *Plasmodium falciparum* accounts for over 90% of malaria cases in Burkina Faso, with the remainder attributable to *Plasmodium malariae* and *Plasmodium ovale*. The primary malaria vector throughout the country is *Anopheles gambiae *sensu lato (*s.l*.), with *Anopheles coluzzii* predominating in the North and *An. gambiae *sensu stricto (*s.s*.) in the Sud-Ouest [27]. The rainy season is when mosquito population density is at its highest. This season typically lasts from May to October in the Boucle du Mouhoun region, and from April to October in the Sud-Ouest region.

SMC campaigns have been conducted annually in various districts of Burkina Faso since 2014, targeting all children under 5 years of age in four to five cycles spaced one month apart, from June through October. The Boucle du Mouhoun region received SMC starting in 2016, and the Sud-Ouest received SMC starting in 2018. Both regions continued SMC activities annually between July and October through 2021. SMC coverage rates were consistently reported above 100%, with little geographic variation [13, 28].

Malaria Indicator Surveys (MIS) conducted in 2014 [29] and 2017–2018 [26], as well as the 2021 Demographic Health Survey (DHS) found that ITN ownership and use were consistently lower in the Sud-Ouest compared to the Boucle du Mouhoun. In 2021, 49.5% of households in Boucle du Mouhoun reported at least one household member sleeping under an ITN the previous night, compared to 37.7% in the Sud-Ouest. Similarly, the percentage of households possessing at least one ITN was 89.7% in Boucle du Mouhoun compared with 78.2% in the Sud-Ouest [30].

Mass distributions of ITNs took place in 2016 and again in October 2019. During the 2019 campaign standard pyrethroid ITNs were distributed in the four study districts, with over 98% of households reported receiving an ITN in all four districts. Following the distribution, the 2021 DHS found that net access increased to 90% in the Boucle du Mouhoun, and to 78% in the Sud-Ouest [30]. Cross-sectional surveys conducted annually from 2019 to 2021 by the New Nets project reported household ownership ranging from 57 to 75%, and ITN use ranging from 21 to 44% in Gaoua in the Sud-Ouest (these survey data were not available for sites in the Boucle du Mouhoun) [31].

### Intervention

From 2018 to 2021, PMI VectorLink deployed annual IRS spray campaigns in Solenzo and Kampti. Neither district had received IRS prior to 2018. IRS campaigns were implemented in June of each year, prior to the onset of the rainy season, rotating three long-lasting non-pyrethroid insecticide products: pirimiphos methyl (Actellic^™^ 300CS), clothianidin (SumiShield^®^ 50WG), and a combination product of deltamethrin (a pyrethroid) with clothianidin (Fludora^™^ Fusion). In Solenzo, both clothianidin and pirimiphos methyl products were deployed in 2018; and only clothianidin was deployed in 2019 and 2020. In 2021, clothianidin along with combination deltamethrin and clothianidin products were deployed in Solenzo. In Kampti, clothianidin was deployed in 2018; a combination clothianidin and deltamethrin in 2019 and 2020; and pirimiphos methyl in 2021 [32–35]. The results of insecticide residual efficacy testing conducted after each IRS campaign yielded high mortality rates (> 80%) for a minimum of eight months following spray with any insecticide product [36].

Insecticide susceptibility testing carried out by the PMI VectorLink project in collaboration with the *Institut de Recherche en Sciences de la Santé* (IRSS) has detected vector resistance to pyrethroids at sentinel sites nationwide, though vectors continue to be susceptible to non-pyrethroids, including pirimiphos-methyl and clothianidin [36, 37]. Entomological monitoring efforts have found that the indoor resting density of *An. gambiae s.l*., measured monthly in the six months post-IRS each year, was significantly lower in Kampti than in a comparator district, Gaoua, after both campaigns. Differences in post-IRS indoor resting density between Solenzo and its comparator district Nouna, were not significant [36–39].

IRS campaign data were obtained from PMI VectorLink end-of-spray reports [32–35]. IRS spray coverage was calculated as the number of structures sprayed divided by the number of structures found.

### Study design

This study employed a retrospective quasi-experimental design using routine surveillance data to assess the impact of the 2018, 2020, and 2021 IRS campaigns on population-adjusted laboratory-confirmed uncomplicated malaria cases in Solenzo and Kampti. The 2019 IRS campaign was not included in this study due to a health sector strike in 2019 that disrupted routine data reporting. Solenzo and Kampti were each compared against a control district selected based on similar geographic area and observed malaria epidemiology. Nouna served as the control district for Solenzo in the Boucle du Mouhoun region; and Gaoua served as the control district for Kampti in the Sud-Ouest region (Fig. 1). The study period encompassed July 2016 to June 2022, giving a baseline of 24 months before the first IRS campaign in 2018, and extending to 12 months after the 2021 IRS campaign.

The primary outcome measure was confirmed uncomplicated malaria cases, adjusted for estimated health facility catchment population (see below for population estimation methods). In Burkina Faso, confirmed malaria cases are classified based on a positive laboratory test for malaria (rapid diagnostic test (RDT) or microscopy); and clinical presentation of fever and/or other symptoms consistent with malaria. These data are reported through the national electronic routine surveillance reporting system, *Entrepôt de données sanitaires du Burkina Faso* (ENDOS). Monthly malaria case data were obtained upon request from the Burkina Faso NMCP for all health facilities in the four study districts.

Health facilities that reported incomplete data for three or more months in any 12-month malaria transmission year (defined as July to June, to capture seasonal malaria transmission peaks) were excluded from the analysis. Incomplete data was determined when a health facility reported a missing value for confirmed malaria cases and/or a non-zero value for clinically diagnosed malaria cases, which indicates that the number of confirmed cases during that month was underestimated. Outlying values, defined as greater than five standard deviations from the mean non-zero value reported by that health facility between January 2016 and June 2022, were reviewed and replaced with null (missing) values. Private and other non-public health facilities were also excluded due to potential overlap in catchment populations with public health facilities.

### Estimating catchment populations

Population data is not reported at the level of health facility catchment areas in Burkina Faso. In order to calculate health facility-level incidence rates, populations were estimated using a catchment model based on travel time to health facilities [40]. The four study districts were grouped by region (Nouna and Solenzo in the Boucle de Mouhoun region; Kampti and Gaoua in the Sud-Ouest region) and divided into a grid of approximately 1 km by 1 km pixels. The estimated travel times for individuals in each pixel to attend each health facility was calculated using a friction surface developed by Weiss et al. [41]. The health facilities included in the model were all those listed in ENDOS which were geo-located and could be considered “active”, i.e. reporting any malaria case data or outpatient data during the study period. The model was run for each calendar year from 2016 to 2022 based on the health facilities identified as active for that year. The catchment model used a population surface from the GRID3 project [42] as a basis for populations in 2019, and projected forward to 2022 and backward to 2016 assuming a 3% growth rate. Catchments were weighted using average annual non-malaria outpatient visits as reported to ENDOS by each health facility, and bounded using district-level populations from the 2019 Burkina Faso census [43].

### Seasonal variables

Monthly precipitation data were pulled from the Climate Hazards Group InfraRed Precipitation with Station (CHIRPS) dataset [44] and monthly enhanced vegetation index (EVI) data were pulled from the Famine Early Warning Systems Network (FEWS NET) [45]. Data were transformed using Alteryx software to be matched spatially and temporally to the primary data sources in this analysis [46]. The final climate dataset included total monthly precipitation and EVI averaged over the area of each commune (the administrative level below district). Precipitation was included in the final model with a two-month lag, and EVI was included with a one-month lag. Both lagged climate covariates were scaled to have a mean of zero and standard deviation of one.

### Estimating malaria incidence rates

An interrupted time-series analysis with control (ITSc) method was used to detect whether there was a change in level and trajectory of malaria case incidence following each IRS campaign. The model used total confirmed uncomplicated malaria cases as the dependent (outcome) variable and used an offset of estimated health facility catchment population. A generalized estimating equation model was fit to control for correlation within each health facility catchment area. A negative binomial distribution was selected to account for hypothesized overdispersion in the data, and a first-order autoregressive correlation structure was specified to account for linearly associated correlation between repeated measures from the same facility over time. Seasonality was controlled for using lagged climate variables (precipitation and EVI) and a dummy variable corresponding to regional high season to adjust for seasonality not accounted for by precipitation and EVI alone (e.g., elevated parasite prevalence). Because of both hypothesized and observed differences in regional contextual factors potentially associated with the impact of IRS on local malaria activity, statistical analyses were stratified by geographic region (Boucle du Mouhoun and Sud-Ouest).

The IRS intervention time points were set at July of each year 2018, 2020, and 2021. Separate models were fitted for each IRS intervention period and stratified by region, for a total of six models. In each model the 12 months following each IRS campaign was compared to the 24-month pre-IRS baseline period. Following the 2018 IRS campaign, the post-IRS period encompassed only 11 months due to a disruption in reporting of routine data that occurred from June to November 2019 as part of a national health sector strike in Burkina Faso. The post-intervention periods in 2020 and 2021 encompassed 12 months. Thus, the baseline period of July 2016 to July 2018 was compared to (1) July 2018 to May 2019; (2) July 2020 to June 2021; and (3) July 2021 to June 2022. Periods were compared to each other within each district, and to the simultaneous periods between IRS and matched non-IRS control districts. Full model specification is further detailed in the supplemental material.

All covariates included in the final model were assessed individually in a univariate model which controlled for time and for repeated measures within health facilities. A full model was then run to examine the change in malaria case incidence following IRS after adjusting for annual and seasonal trends, climate, and clustering of observations by health facility catchment area. Calculated incidence rate ratios (IRR) with associated confidence intervals (CI) were determined to be statistically significant if found to have a p-value < 0.05.

Using the modeled case estimates, percent change in case incidence from the 24-month pre-IRS baseline period to the post-IRS period was calculated by dividing the sum of all estimated cases during the post-IRS period by the sum of all estimated cases during the baseline period.

## Results

### IRS campaign coverage

**Fig. 2:**
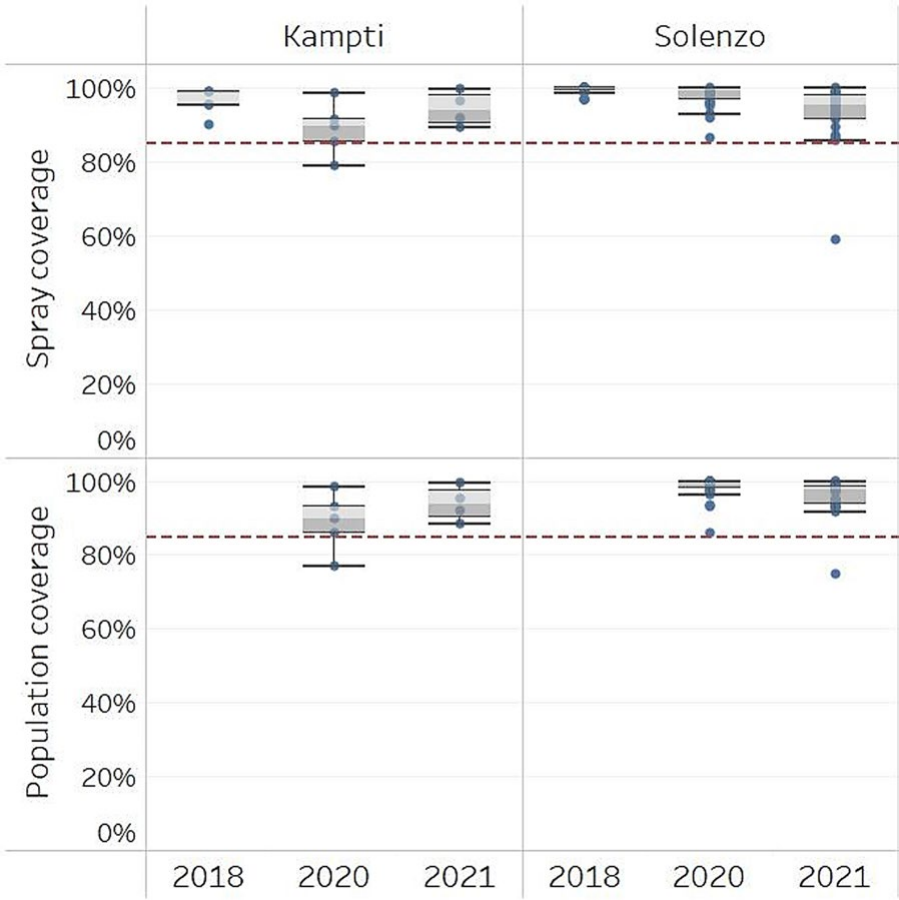
Boxplots of population and spray coverage by facility catchment area achieved during the 2018, 2020, and 2021 IRS campaigns in Kampti and Solenzo districts. The horizontal dashed line indicates the 85% coverage target recommended by the WHO. Spray coverage is calculated as the number of structures sprayed divided by the number of structures found. Population coverage is calculated as the population protected divided by the sum of the population protected and the population not protected. Population and spray coverage data were collected by spray teams during the campaign. Population coverage data was not available from the 2018 campaign

Among facility catchment areas included in this study, a total of 80,708 structures were sprayed in the 2018 IRS campaign; 123,213 in 2020; and 99,199 in 2021, across both IRS districts. District-level spray coverage exceeded 90% in both Solenzo and Kampti during the 2018 and 2021 IRS campaigns. In 2020, IRS coverage in Kampti dipped to 89%, largely due to difficulty gaining acceptance in peri-urban areas [47]. Coverage in Solenzo remained high that year at 97%. Health facility catchment-level coverage data shows that all catchment areas in Solenzo achieved greater than 85% coverage (target coverage defined by WHO guidelines [3]) across all 3 years, except for one facility catchment area where spray coverage was 59% in 2021. Similarly, in Kampti, only one facility catchment area achieved less than 85% spray coverage in 2020, with all other catchment areas exceeding the target across the three campaigns. A total of 385,441 people were protected by IRS campaigns in 2020; and 331,405 in 2021 across both districts. Population coverage exceeded 85% in all facility catchment areas except for one in Kampti in 2020 and one in Solenzo in 2021. Population coverage data were not available from the 2018 campaign. District-level IRS coverage is presented in Table 1, and facility-catchment level coverage is presented in Fig. 2.

**Table 1:** Number of health facilities and monthly health facility records from Burkina Faso’s ENDOS routine surveillance system which were included in the analysis, July 2016 to June 2022; along with IRS coverage indicators for the 2018, 2020, and 2021 IRS campaigns in Solenzo and Kampti

		Data Inclusion					IRS Coverage		
Region	District	Facilities identified in ENDOS	Facilities meeting inclusion criteria (%)	Facility-months included in each model (%)			Structures sprayed/structures found (%) Population protected/population found (%)		
				2018	2020	2021	2018	2020	2021
Boucle du Mouhoun	Solenzo (IRS)	41	31 (76%)	1078 (99%)	1,111 (99%)	1,102 (99%)	79 461/79 918 (99%)	114 283/116 961 (98%) 354 998/360 400 (99%)	92 370/99 798 (93%) 304 659/317 755 (96%)
	Nouna (control)	55	29 (53%)	912 (90%)	948 (91%)	946 (91%)	–	–	–
Sud Ouest	Kampti (IRS)	16	5 (31%)	156 (89%)	161 (89%)	160 (89%)	10 247/10 566 (97%)	8 930/10 022 (89%) 30 443/34 136 (88%)	6 829/7 242 (94%) 26 746/28 590 (94%)
	Gaoua (control)	52	27 (52%)	926 (98%)	946 (97%)	955 (98%)	–	–	–

Population and structure coverage data were collected by spray teams during the campaign. Population coverage data was not available from the 2018 campaign

### Catchment populations

Of the 164 health facilities identified in ENDOS during the evaluation period, 129 were eligible for inclusion in catchment population modelling, based on reporting outpatient and/or malaria case data during each year of the evaluation period (considered “active”) and being geo-located. In 2016, 121 facilities were considered active; 124 in 2017; 129 in 2018; 128 in 2019; 128 in 2020; 128 in 2021; and 125 in 2022. Of the facilities included in catchment population modelling, 40 health facilities were in Solenzo; 47 in Nouna; 12 in Kampti; and 30 in Gaoua.

**Fig. 3:**
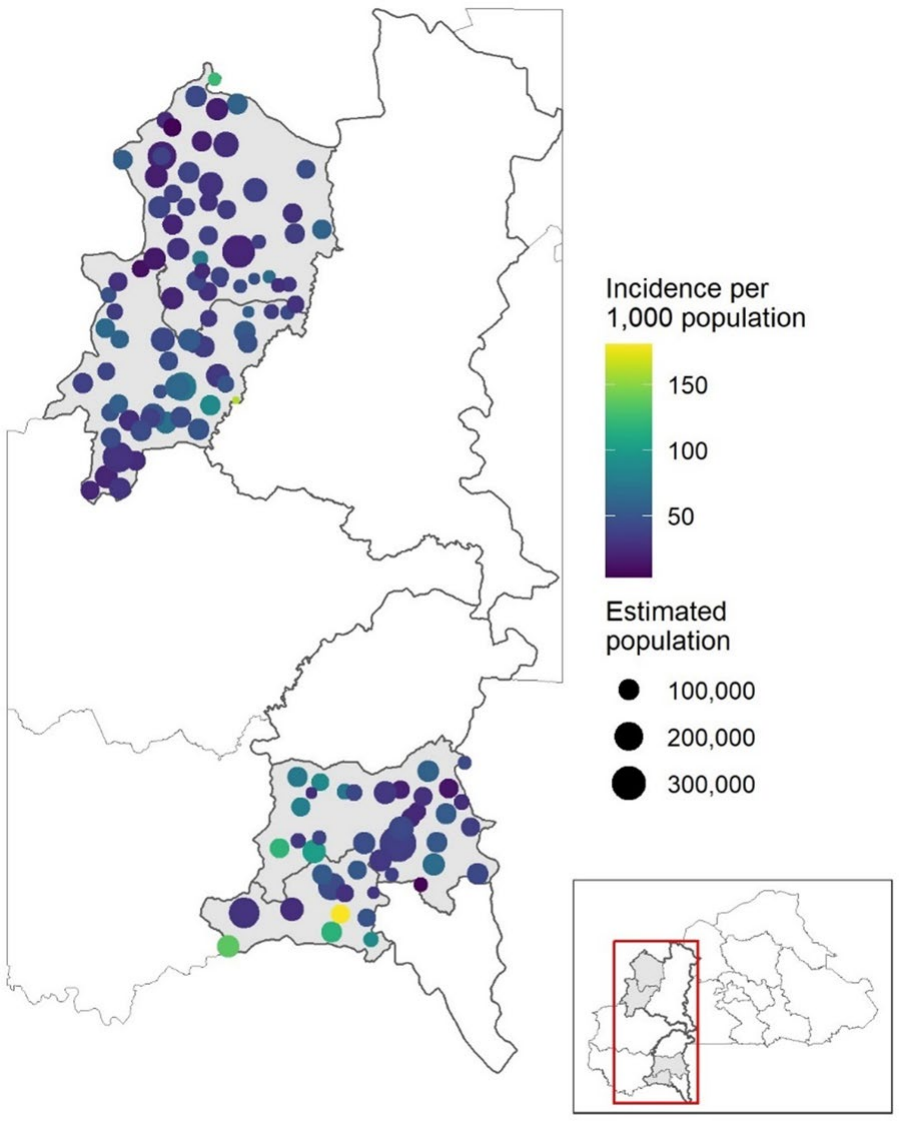
Modelled catchment population sizes and annual malaria case incidence per 1000 estimated population for 144 health facilities in Boucle du Mouhoun and the Sud-Ouest regions. Population and incidence estimates shown are for the year 2018

The median modeled catchment population was 7,022 (LQ = 5,277, UQ = 8,935). The total population served across the four study districts according to the model grew from 866,038 in 2016 to 1,062,497 in 2022, which is largely aligned with the census population of 1,002,321 from 2019 [43]. Annual malaria case incidence per 1000 estimated population at the health facilities included in catchment modelling are shown in Fig. 3.

### Health facility surveillance data cleaning and inclusion

**Fig. 4:**
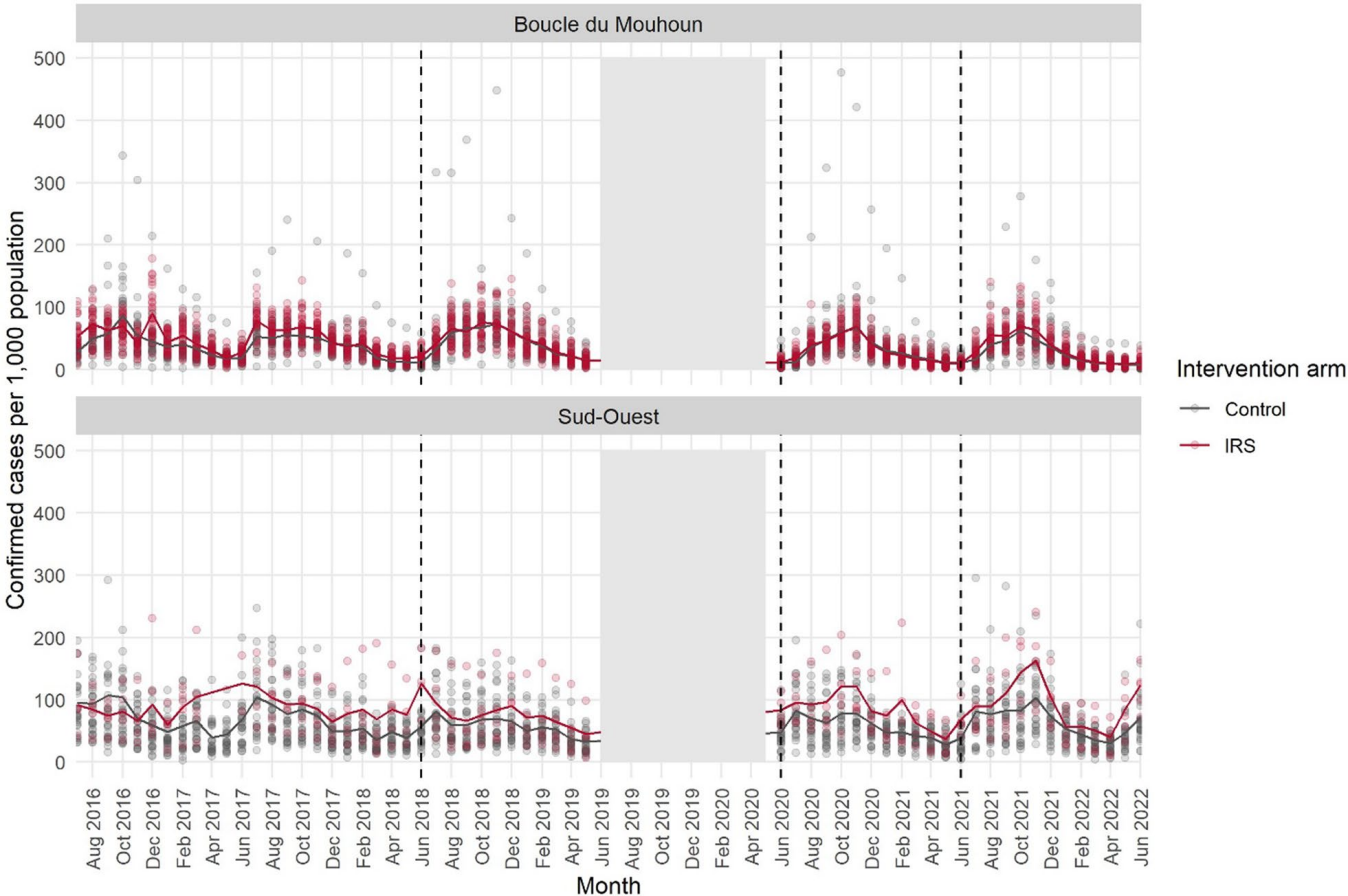
Confirmed malaria cases per 1000 estimated population from July 2016 to June 2022. Solid lines indicate incidence at the district level, and points represent values reported by each facility. The dates of the 2018, 2020, and 2021 IRS campaigns are indicated by black dashed lines, and the grey bands indicate the period of data omitted from June 2019 to May 2020 due to a health sector strike and data unavailability

This analysis included 91 health facilities which met the inclusion criteria out of the total 164 health facilities identified in the four study districts. A total of 5300 (99%) monthly records from these health facilities were included in the statistical model (Table 1). Six records containing outlying values were determined to be data entry errors and designated as missing data. A total of 1,878,232 confirmed uncomplicated malaria cases were reported during the study period months between July 2016 and June 2022 (excluding June 2019–June 2020) from the health facilities included in this analysis, of which 793,416 (42.2%) were reported from facilities in IRS districts. Figure 4 displays monthly-confirmed malaria cases per 1000 estimated population in the four study districts.

### Estimated malaria case rates

**Fig. 5:**
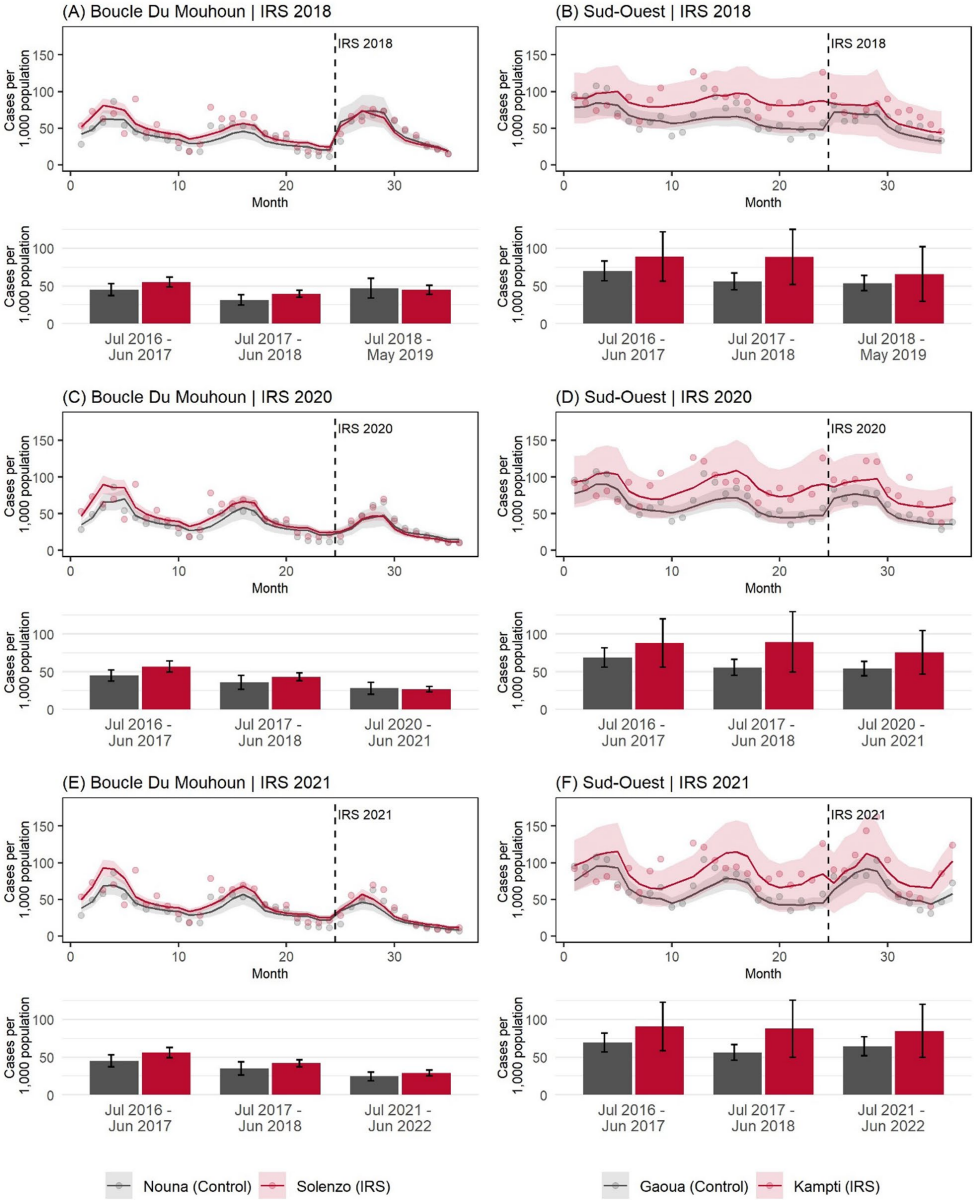
Model-predicted confirmed malaria cases per 1,000 estimated population in the Boucle du Mouhoun and Sud-Ouest regions. In the time-series line graphs, model predictions are represented by solid lines and observed values are represented by points. Vertical dashed lines indicate the conclusion of IRS campaigns in June 2018, 2020, and 2021. The x-axis represents months from the start of the study. The pre-IRS baseline period is July 2016 to June 2018 for all plots. The post-IRS period for plots (**A**) and (**B**) is July 2018 to May 2019. The post-IRS period for plots (**C**) and (**D**) is July 2020 to June 2021. The post-IRS period for plots (**E**) and (**F**) is July 2021 to June 2022. Bar charts display modelled annualized case incidence. IRS districts are colored red and control districts are in grey

Incidence rate ratios for all model covariates are presented in Table 2, and Fig. 5 displays predicted confirmed malaria cases per 1,000 estimated population plotted with observed values for all fitted models.

**Table 2:** Multivariate incidence rate ratios (IRR) for all-ages laboratory-confirmed uncomplicated malaria cases reported through routine surveillance, adjusted for estimated catchment population in the Boucle du Mouhoun region

	2018 IRS				2020 IRS				2021 IRS			
	IRR	95% CI		P	IRR	95% CI		P	IRR	95% CI		P
		Lower	Upper			Lower	Upper			Lower	Upper	
IRS exposure												
Unsprayed districts	Ref				Ref				Ref			
Sprayed districts	1.138	0.931	1.392	0.207	1.178	0.970	1.431	0.098	1.159	0.947	1.418	0.152
IRS districts												
Pre-intervention monthly trend in population-adjusted all-ages malaria cases^a^	0.974	0.969	0.979	< 0.001**	0.979	0.974	0.984	< 0.001**	0.978	0.973	0.983	< 0.001**
Post-intervention change in monthly trend of population-adjusted all-ages malaria cases^b^	0.933	0.923	0.944	< 0.001**	0.958	0.943	0.973	< 0.001**	0.932	0.916	0.949	< 0.001**
Post-intervention change in level of population-adjusted all-ages malaria cases^b^	1.942	1.746	2.160	< 0.001**	0.908	0.786	1.049	0.192	1.222	1.059	1.410	0.006*
Control districts												
Pre-intervention monthly trend in population-adjusted all-ages malaria cases^a^	0.972	0.965	0.979	< 0.001**	0.982	0.970	0.993	0.002	0.981	0.972	0.990	< 0.001**
Post-intervention change in monthly trend of population-adjusted all-ages malaria cases^b^	0.924	0.898	0.951	< 0.001**	0.974	0.950	0.997	0.029*	0.900	0.868	0.933	< 0.001**
Post-intervention change in level of population-adjusted all-ages malaria cases^b^	2.844	2.429	3.330	< 0.001**	1.092	0.971	1.229	0.140	1.416	1.199	1.671	< 0.001**
Difference between IRS and control districts												
Pre-intervention difference in monthly trend of population-adjusted all-ages malaria cases^a^	1.002	0.993	1.011	0.627	0.997	0.984	1.010	0.653	0.997	0.987	1.007	0.576
Post-intervention difference in change in monthly trend of population-adjusted all-ages malaria cases^b^	1.010	0.978	1.042	0.545	0.984	0.955	1.014	0.292	1.036	0.994	1.079	0.095
Post-intervention difference in change in level of population-adjusted all-ages malaria cases^b^	0.683	0.564	0.827	< 0.001**	0.831	0.687	1.006	0.057	0.863	0.698	1.069	0.177
Control variables												
Rainfall (lagged 2 months), per 10 mm	1.000	0.947	1.057	0.988	0.933	0.865	1.007	0.074	1.050	1.001	1.102	0.045*
EVI (lagged 1 month)	1.258	1.189	1.332	< 0.001**	1.459	1.344	1.585	< 0.001**	1.284	1.214	1.358	< 0.001**
High season (binary)	0.858	0.831	0.885	< 0.001**	0.817	0.794	0.840	< 0.001**	0.854	0.831	0.878	< 0.001**
Observations	1.990				2.052				2.048			
Number of health facilities	59				59				59			

*P < 0.05; **P < 0.001

^a^The baseline period comprises July 2016 to June 2018

^b^The post-IRS period comprises July 2018 to May 2019 for the 2018 IRS evaluation; July 2020 to June 2021 for the 2020 IRS evaluation; and July 2021 to June 2022 for the 2021 IRS evaluation

#### Boucle du Mouhoun

All three models estimated that the monthly trend in population-adjusted confirmed malaria cases was decreasing significantly in both the control district Nouna (Model 1: IRR = 0.972, 95% CI 0.965–0.979; Model 2: IRR = 0.982, 95%CI = 0.970–0.993; Model 3: IRR = 0.981, 95% CI 0.972–0.990) and the IRS district Solenzo (Model 1: IRR = 0.947, 95% CI 0.969–0.979; Model 2: IRR = 0.979, 95% CI 0.974–0.984; Model 3: IRR = 0.978, 95% CI 0.973–0.983) over the 24-month pre-IRS baseline period from July 2016 to June 2018. The baseline trends in the two districts were not found to be significantly different from one another in all three models (P > 0.05).

In the month immediately following the 2018 IRS campaign, malaria rates increased significantly in the control district (Nouna) by 184.4% (IRR = 2.844; 95% CI 2.426–3.330) and by 94.2% (IRR = 1.942; 95% CI 1.746–2.160) in the IRS district (Solenzo), compared to the month preceding the campaign. The difference in percent change of malaria rates between the two districts was statistically significant (P < 0.001).

The month after the 2020 IRS campaign, there was no significant change in malaria case rates from the previous month in either Nouna (IRR = 1.092; 95% CI 0.971–1.229) or Solenzo (IRR = 0.908; 95% CI 0.786–1.049), and the difference in change between the two districts was not significant at the 5% level (P = 0.057).

In the month after the 2021 IRS campaign, rates in Nouna increased by 41.6% (IRR = 1.416; 95% CI 1.199–1.671) from the previous month. In Solenzo, rates increased by 22.2% (IRR = 1.222; 95% CI 0.059–1.410). The difference in change between the two districts was not statistically significant (P > 0.05).

Both districts observed changes in monthly malaria rate trends following IRS campaigns. These changes were slight (< 10%), and there was no significant difference in change observed between IRS and control districts (P > 0.05).

#### Sud-Ouest

All three ITS models estimated that the baseline (July 2016–June 2018) monthly trend in population-adjusted confirmed malaria cases was decreasing in the control district Gaoua (Model 1: IRR = 0.983, 95% CI 0.977–0.990; Model 2: IRR = 0.985, 95% CI 0.979–0.991; Model 3: IRR = 0.986, 95% CI 0.980–0.992). The baseline trend in the IRS district Kampti was not found to be statistically significantly different from Gaoua in the three models (P > 0.05).

In the Sud-Ouest, malaria case incidence immediately following all three IRS campaigns was found to decrease significantly in Kampti compared to the baseline and/or to the control district Gaoua.

Following deployment of IRS in 2018, malaria rates in Gaoua increased by 43.4% (IRR = 1.434; 95% CI 1.231–1.670) compared to the month preceding the campaign. In the IRS district Kampti, malaria rates decreased by 8.4% (IRR = 0.916; 95% CI 0.849–0.978). The difference in percent change of malaria rates between Kampti and Gaoua was statistically significant (P < 0.001).

After the 2020 IRS campaign, malaria case rates in Gaoua increased by 36.6% (IRR = 1.366; 95% CI 1.174–1.590) from the baseline period. In Kampti, the increase in malaria case rates post-IRS was significantly less than in Gaoua (IRR = 0.638; 95% CI 0.441–0.924), although case rates did not change significantly when compared to the baseline period (IRR = 0.872; 95% CI 0.627–1.212).

After the 2021 IRS campaign, case rates in Gaoua increased by 23.2% (IRR = 1.232; 95% CI 1.065–1.425) from the baseline period. In Kampti, case rates increased significantly less than in Gaoua (IRR = 0.619; 95% CI 0.418–0.918), although they did not change significantly compared to the pre-IRS period (IRR = 0.763; 95% CI 0.532–1.095).

Following IRS in 2018 both Kampti and Gaoua observed slightly decreasing trends in monthly malaria rates. These changes were less than 10%, and there was no significant difference between IRS and control districts (P > 0.05). Following the 2021 IRS campaign, a slightly increasing trend was observed in both districts, although it was not significant and there was no significant difference between IRS and control districts (P > 0.05).

Incidence rate ratios for all Sud-Ouest model covariates are presented in Table 3, and Fig. 5 displays predicted confirmed malaria cases per 1000 estimated population plotted with observed values for all fitted models.

**Table 3:** Multivariate incidence rate ratios (IRR) for all-ages laboratory-confirmed uncomplicated malaria cases reported through routine surveillance, adjusted for estimated catchment population in the Sud-Ouest region

	2018 IRS				2020 IRS				2021 IRS			
	IRR	95% CI		P	IRR	95% CI		P	IRR	95% CI		P
		Lower	Upper			Lower	Upper			Lower	Upper	
IRS exposure												
Unsprayed districts	Ref				Ref				Ref			
Sprayed districts	1.108	0.716	1.713	0.645	1.095	0.709	1.690	0.683	1.092	0.715	1.670	0.683
IRS districts												
Pre-intervention monthly trend in population-adjusted all-ages malaria cases^a^	1.002	0.981	1.024	0.840	1.005	0.980	1.030	0.700	1.003	0.980	1.026	0.829
Post-intervention change in monthly trend of population-adjusted all-ages malaria cases^b^	0.945	0.918	0.973	< 0.001**	0.979	0.947	1.013	0.218	1.027	0.973	1.084	0.331
Post-intervention change in level of population-adjusted all-ages malaria cases^b^	0.916	0.849	0.978	0.022*	0.872	0.627	1.212	0.415	0.763	0.532	1.095	0.142
Control districts												
Pre-intervention monthly trend in population-adjusted all-ages malaria cases^a^	0.983	0.977	0.990	< 0.001**	0.985	0.979	0.991	< 0.001**	0.986	0.980	0.992	< 0.001**
Post-intervention change in monthly trend of population-adjusted all-ages malaria cases^b^	0.947	0.932	0.963	< 0.001**	0.963	0.947	0.980	< 0.001**	1.008	0.998	1.019	0.126
Post-intervention change in level of population-adjusted all-ages malaria cases^b^	1.434	1.231	1.670	< 0.001**	1.366	1.174	1.590	< 0.001**	1.232	1.065	1.425	0.005*
Difference between IRS and control districts												
Pre-intervention difference in monthly trend of population-adjusted all-ages malaria cases^a^	1.019	0.997	1.042	0.098	1.020	0.994	1.047	0.136	1.017	0.993	1.042	0.174
Post-intervention difference in change in monthly trend of population-adjusted all-ages malaria cases^b^	0.998	0.965	1.031	0.891	1.016	0.979	1.055	0.397	1.019	0.964	1.076	0.508
Post-intervention difference in change in level of population-adjusted all-ages malaria cases^b^	0.639	0.537	0.760	< 0.001**	0.638	0.441	0.924	0.017*	0.619	0.418	0.918	0.017*
Control variables												
Rainfall (lagged 2 months), per 10 mm	1.058	1.013	1.104	0.010*	1.053	1.021	1.086	0.001*	1.034	1.004	1.065	0.025*
EVI (lagged 1 month)	1.055	0.999	1.113	0.052	1.132	1.084	1.183	< 0.001**	1.255	1.194	1.320	< 0.001**
High season (binary)	0.954	0.909	1.002	0.061	0.962	0.919	1.007	0.097	0.873	0.832	0.916	< 0.001**
Observations	1.082				1.107				1.115			
Number of health facilities	32				32				32			

*P < 0.05; **P < 0.001

^a^The baseline period comprises July 2016 to June 2018

^b^The post-IRS period comprises July 2018 to May 2019 for the 2018 IRS evaluation; July 2020 to June 2021 for the 2020 IRS evaluation; and July 2021 to June 2022 for the 2021 IRS evaluation

Across both Boucle du Mouhoun and Sud-Ouest region models, the high season variable appeared to have a negative association with malaria rates. In the Sud-Ouest, the association was only significant in the model evaluating the 2021 IRS campaign, where high season months were significantly associated with a 12.7% decrease in malaria rates (IRR = 0.873; 95% CI 0.832–0.916). In the Boucle du Mouhoun, high season was significantly associated with decreases of 14.2% in the first model (IRR = 0.858; 95% CI 0.831–0.885), 18.3% in the second model (IRR = 0.817; 95% CI 0.794–0.840), and 14.6% in the third model (IRR = 0.854; 95% CI 0.831–0.878).

## Discussion

The results presented here suggest a positive impact of IRS in the Sud-Ouest region following campaigns in 2018, 2020, and 2021. In the IRS district Kampti, rates of routinely reported confirmed malaria case incidence were between 36 and 38% lower than in the control district following each intervention. In the Boucle du Mouhoun region, IRS appeared to confer diminishing impact with each successive campaign. Following the 2018 IRS campaign, malaria rates in Solenzo were 31.7% lower than in the control district Nouna. After the 2020 campaign, rates in Solenzo were 16.9% lower than in Nouna, and the difference was just above the 5% significance threshold (P = 0.057). No significant impact was detected following the 2021 IRS campaign. The percent reductions in modelled malaria case incidence from the baseline period to the post-IRS period was greater in Solenzo (IRS) than in Nouna (control) for all three campaigns, although the statistical significance of this difference was not determined.

Reported IRS structure coverage and population coverage in Solenzo was notably higher than coverage in Kampti in the 2020 campaign. However, coverage across both districts was consistently above the recommended WHO threshold of 85%, and it is unlikely that slight differences in high coverage levels would have caused the differences in estimated impact between the districts. In Kampti, IRS structure and population coverage dipped to 89 and 88%, respectively, in 2020 and a single health facility catchment area did not achieve > 85% coverage that year. However, the estimated impact of IRS across the three campaigns does not appear to reflect these year-to-year variations in coverage.

The IRS interventions studied here took place alongside two other concomitant high-impact anti-malaria interventions (ITN distributions and annual SMC campaigns), which limits the ability of this study to discern whether IRS independently caused any of the observed effects. In the Boucle du Mouhoun region, SMC campaigns began in 2016, around the start of the study period for this analysis and occurred annually through 2021. In the Sud-Ouest, SMC implementation began in 2018, the same year as the first IRS campaign, and continued annually through 2021. SMC campaigns in Burkina Faso are implemented each year from June or July to October, which is the period directly after IRS campaigns take place, making it difficult to temporally distinguish the independent effect of either intervention in the absence of an additional control district that received IRS but not SMC. SMC implementation was part of the baseline period in the Boucle du Mouhoun region, meaning that the effect of each IRS campaign is being measured against a period of potentially already reduced malaria incidence, which may obscure any added benefit of IRS. In the Sud-Ouest, SMC implementation was not part of the baseline period but was implemented each year directly after the IRS campaigns. Thus, the estimated impact from these models, which compare post-IRS periods against the baseline period, is likely reflecting a combined IRS and SMC effect.

SMC was previously found to have a significant protective effect in several districts of Burkina Faso [48–50], and other studies have detected a possible combined effect for the co-implementation of IRS and SMC implemented over one to two years in Mali [19] and in Senegal [20]. However, the longer-term effect of sustained SMC and its interaction with IRS is less well-studied.

The observable impact of IRS was likely also influenced by the October 2019 ITN campaign in which all study districts (both IRS and control districts) received new standard pyrethroid ITNs. As reported in the most recent MIS and DHS surveys from 2014, 2017–2018, and 2021, the Sud-Ouest region consistently reports lower ITN use and household ownership than the Boucle du Mouhoun region [26, 30, 31]. IRS may have a greater impact in areas where there is lower population use of ITNs, which could explain why a beneficial impact was detected in Kampti and not in Solenzo, even though IRS coverage was lower in Kampti compared to Solenzo.

The two IRS districts included in this study are located in two different climate zones (tropical vs. Sudano-Sahelien) with differences in the seasonality of malaria transmission. The effect of IRS and its combined implementation with other malaria control interventions across different settings is not well understood, and it may be reasonable to expect a differential impact in varying contexts. Further studies comparing intervention effectiveness in different malaria transmission zones may help to elucidate the key factors that would impact the timing and targeting of combined malaria control interventions relative to one another and to transmission seasons.

The model showed that high season months were independently significantly associated with a decrease in population-adjusted malaria cases, which is counter-intuitive (one would expect high season months to be associated with higher malaria rates due to factors such as higher underlying parasite prevalence in the population). A reasonable interpretation of this result is that, after adjusting for climate variables—which were otherwise highly correlated with malaria rates and generally peak during the high season months—the number of observed cases was lower than the model would have predicted. This could either be due to the model’s overreliance on EVI as a predictor of malaria rates or related to the impact of the annual SMC campaigns which coincided with the high season months (July to October).

Selecting an appropriate population offset by which to adjust crude malaria case counts was a challenge in the absence of available health facility catchment populations. The model used for estimating these catchment populations is based primarily on travel time and may not account for other factors that may influence catchment population size. The potential influence of differential treatment-seeking rates was accounted for by using non-malaria outpatient visits to weight the estimates. Additionally, only populations for the four study districts were included in population modelling, which does not account for cases which may be imported from neighbouring districts. However, as health facility-level populations are often unavailable from routine sources, this method of modelling catchment populations permitted a robust analysis at a granular level that would have otherwise been infeasible.

Low levels of health facility reporting completeness meant that 37% of facilities in IRS districts and 48% of facilities in control districts were excluded from this analysis. Kampti was particularly impacted, with only five health facilities (31% of facilities) meeting the eligibility criteria. Exclusion of this data potentially hindered the representativeness of the sample used in the study if the included and excluded health facilities were systematically different from one another in the indicators measured. The Sud-Ouest analysis in particular may have been underpowered to detect the true impact of IRS.

Finally, the primary indicator used in this evaluation to measure malaria activity was confirmed malaria cases of patients who sought care at health facilities, reported through Burkina Faso’s electronic routine data reporting system. This can introduce bias if there are overall differences between IRS and non-IRS districts in health-seeking behaviour, availability of drugs and RDT kits, or data reporting [51].

Despite these limitations, entomological monitoring results suggest some alignment with the results observed in this study. A significant difference in post-IRS indoor resting density between the Sud-Ouest IRS district Kampti and its control district Gaoua was observed following both the 2018 and 2020 IRS campaigns. However, no such difference in post-IRS indoor resting density was detected between the IRS and control districts in the Boucle du Mouhoun either year [36, 39]. These entomological indicators support this study’s findings of a differential impact of IRS on malaria transmission in the two distinct settings.

## Conclusions

As national malaria programmes seek to improve targeting of malaria control interventions to areas where they will achieve the greatest impact, there is a need for evidence around the effectiveness of these interventions both alone and in combination, in various malaria transmission contexts. In this study, routine case data and modelled health facility catchment populations were used to assess the impact of IRS campaigns in 2018, 2020 and 2021 on population-adjusted malaria cases in two regions of Burkina Faso. This study points to a differential effect of IRS in the tropical region, where a positive impact of IRS was detected, versus the Sudano-Sahelien region where an increase in malaria case incidence was detected following IRS. These results suggest that the interaction of IRS with other malaria control interventions (ITN distribution and SMC campaigns) may also differ significantly by context.

### Supplementary Information

Supplementary Material 1.

## Data Availability

All data are available from the authors upon reasonable request.

## Abbreviations

CHIRPS: Climate Hazards Group InfraRed Precipitation with Station
ENDOS: Entrepôt de Données Sanitaires du Burkina Faso
EVI: Enhanced vegetation index
FEWS NET: Famine early warning systems network
HBHI: High burden to high impact
IRR: Incidence rate ratio
IRS: Indoor residual spraying
ITN: Insecticide-treated net
ITSc: Interrupted time-series with control
MIS: Malaria indicator survey
NMCP: National malaria control programme
PMI: U.S. President’s Malaria Initiative
RDT: Rapid diagnostic test
SMC: Seasonal malaria chemoprevention
