# Supplementary material for: An observational analysis of the impact of indoor residual spraying in two distinct contexts of Burkina Faso
Source: Malar J. 2024 Aug 2;23:229. doi: 10.1186/s12936-024-05054-2 (PMC11295511; doi:10.1186/s12936-024-05054-2)
Supplement: Supplementary file 1 — Supplementary Material 1. [file 12936_2024_5054_MOESM1_ESM.docx]

An Observational Analysis of the Impact of Indoor Residual Spraying in Two Distinct Contexts of Burkina Faso – Supplemental Materials

# 1. Interrupted time series models

Specification of the six interrupted time series models fitted to estimate impact of IRS campaigns is presented below. For each model, the pre-IRS baseline period was July 2016 to June 2018. The post-IRS period was July 2018 to May 2019 for models evaluating the 2018 IRS campaigns; July 2020 to June 2021 for models evaluating the 2020 IRS campaigns; and July 2021 to June 2022 for models evaluating the 2021 IRS campaigns.

$$Y_{t}= \beta_{0}+ \beta_{1}T_{t}+ \beta_{2}X_{t}+ \beta_{3}X_{t}T_{t}+ \beta_{4}Z+ \beta_{5}ZT_{t}+ \beta_{6}ZX_{t}+ \beta_{7}ZX_{t}T_{t}$$

- $Y_{t}$ : Outcome (reported confirmed uncomplicated malaria cases by facility and month).
- $T_{t}$ : Time in months elapsed since the start of the study (first month coded as 1).
- $Z$ : Binary variable to denote assignment of IRS or control (coded as 1 for facilities in Kampti and Solenzo; coded 0 for facilities in Gaoua and Nouna).
- $X_{t}$ : Binary variable for the pre- vs. post-IRS intervention (coded as 0 for pre-IRS months; coded as 1 for post-IRS months).
- $\beta_{0}$ : Intercept at T=0 at control facilities.
- $\beta_{1}$ : Pre-intervention slope at control facilities.
- $\beta_{2}$ : Post-intervention intercept change at control facilities.
- $\beta_{3}$ : Pre- vs. post-intervention change in slope at control facilities.
- $\beta_{4}$ : Difference in intercept at T=0 between intervention and control facilities.
- $\beta_{5}$ : Difference in slope pre-intervention between intervention and control facilities.
- $\beta_{6}$ : Pre- vs. post-intervention difference in intercept change between intervention and control facilities (difference-in-difference of intercepts).
- $\beta_{7}$ : Pre- vs. post-intervention difference in slope between intervention and control facilities (difference-in-difference of slopes).

# 2. Baseline characteristics of the four study districts

|  |  | **Boucle du Mouhoun** | |  | **Sud Ouest** | |
| --- | --- | --- | --- | --- | --- | --- |
|  |  | **IRS**  **Solenzo** | **Control Nouna** |  | **IRS Kampti** | **Control Gaoua** |
| Climate zone |  | Sudano-sahelien | |  | Sudanian | |
|  |  | (semi-arid) | |  | (tropical savannah) | |
|  |  |  |  |  |  |  |
| Annual precipitation, avg 2016 - 2019 (mm) |  | 895 | 789 |  | 1111 | 1124 |
|  |  |  |  |  |  |  |
| Rainy season (avg precipitation >60 mm/month) |  | May - Oct | |  | Apr - Oct | |
|  |  |  |  |  |  |  |
| Lowest elevation^*^ |  | 286 | 254 |  | - | 262 |
| Highest elevation^*^ |  | 368 | 290 |  | - | 448 |
|  |  |  |  |  |  |  |
| District population, 2018 |  | 372 067 | 378 353 |  | 102 800 | 259 841 |
|  |  |  |  |  |  |  |
| Largest urban center |  | Solenzo | Nouna |  | Kampti | Gaoua |
| (population**) |  | (16 850) | (22 166) |  | (2 123) | (25 104) |
|  |  |  |  |  |  |  |
| Annual reported malaria cases |  |  | |  |  | |
| 2017 |  | 181 512 | 161 351 |  | 83 452 | 162 471 |
| 2018 |  | 165 282 | 158 951 |  | 80 513 | 158 857 |
| 2019*^†^* |  | -- | -- |  | -- | -- |
| 2020 |  | 124 098 | 151 524 |  | 91 402 | 170 035 |
| 2021 |  | 135 645 | 144 146 |  | 100 582 | 204 069 |
|  |  |  |  |  |  |  |
| Malaria prevalence in children aged 6 – 59 months by microscopy*** |  |  | |  |  | |
| 2017-2018 |  | 23% | |  | 39% | |
| 2021 |  | 10% | |  | 35% | |

** Source: U.S. Geological Survey; data not found for Kampti areas.*

*** Source: Ministère de l’Economie et des Finances (2009), based on the 2006 national census.*

**** Source: Malaria Indicator Survey, 2017-2018; Demographic Health Survey, 2021*

*^†^ 2019 routine surveillance data is incomplete due to data withholding during nationwide health sector strike*
